# Single-cell analysis of testicular bacterial microbiome changes during aging and effect on reproductive capacity in mice

**DOI:** 10.1016/j.isci.2025.114174

**Published:** 2025-11-22

**Authors:** Jianteng Zhou, Ying Li, Tao Zhu, Kexin Yang, Cheng Zhang, Ruoqi Zhang, Xinrong Zhang, Dianshuang Zhou, Xiaoyue Ding, Yu Qiao, Conghui Han, Zuobin Zhu

**Affiliations:** 1 Jiangsu Engineering Center for Precision Diagnosis and Treatment Research of Polygenic Diseases, Key Laboratory of Genetic Foundation and Clinical Application, Department of Genetics, Xuzhou Medical University, Xuzhou 221004, China; 2 Medical Technology College, Xuzhou Medical University, Xuzhou 221004, China; 3 Center for Reproduction, The Affiliated Huai’an No.1 People’s Hospital of Nanjing Medical University, Huai’an, China; 4 Xuzhou Central Hospital, 199 Jiefang South Road, Xuzhou, Jiangsu, China

**Keywords:** Health sciences

## Abstract

The testis supports spermatogenesis through a tightly regulated microenvironment, and the bacterial microbiome (BM) may influence host cells through immune and metabolic pathways, thereby impacting reproductive capacity. Here, we applied invasion-adhesion-directed expression sequencing (INVADE-seq), a single-cell RNA sequencing approach that simultaneously captures host and bacterial transcripts, to examine how bacterial signals shape testicular cell states. We detected a sparse but widespread bacterial presence across multiple cell types, with somatic and early germ cells outside the blood-testis barrier (BTB) showing relatively higher bacterial abundance. Bacterial load increased with age, coinciding with transcriptional signatures of reduced BTB function. At the cellular level, bacterial-positive Leydig cells exhibited activation of steroidogenic genes, whereas macrophages upregulated pathways related to autophagy and immune modulation. These findings not only deepen our understanding of testicular microbiome biology but also hold promise for the discovery of novel diagnostic biomarkers and therapeutic targets for BM-related and age-associated male subfertility.

## Highlights

• Single-cell INVADE-seq maps bacteria-host interactions across diverse testicular cell types
• Somatic and early germ cells outside the blood-testis barrier show higher bacterial load
• Aging increases testicular bacterial abundance and correlates with impaired BTB integrity
• Bacterial-positive cells activate steroid pathways in Leydig and M2 programs in macrophages

## Graphical abstract

**Figure:**
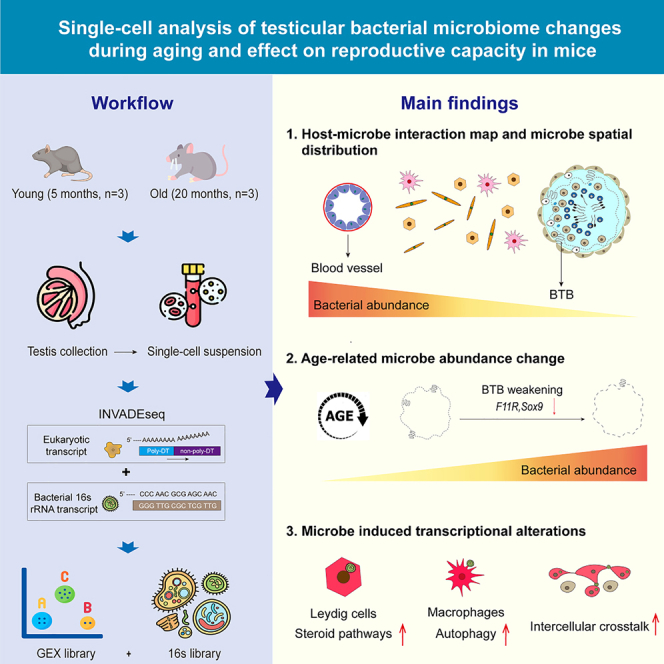

## Introduction

The bacterial microbiome (BM) is ubiquitously distributed across different body regions and plays a pivotal role in modulating host health and development.^1^^,^^2^ The male reproductive system is an intricate and complex system, which is responsible for producing, storing, and transporting sperm, along with the synthesis and release of sexual hormones.^3^ Recent studies have highlighted connections between the BM and male reproductive health. On one hand, gut microbiomes and their metabolites have been shown to influence sperm quality, disrupt testicular architecture, regulate sex hormones, and affect sexual behavior.^4^^,^^5^^,^^6^^,^^7^ On the other hand, the composition and structure of bacterial communities within seminal fluid and the male reproductive tract hold promise as potential biomarkers for assessing sperm quality.^8^^,^^9^^,^^10^ Paradoxically, despite the testis being the primary organ of male reproduction, the BM associated with testicular tissue remains poorly characterized.

Testis is where spermatogenesis takes place. Spermatogenesis represents a complex process, which involves the differentiation of spermatogonia into mature sperm in a stepwise manner. In addition to germ cells at various stages of spermatogenic development, the testicular microenvironment encompasses multiple somatic cell types, including sertoli cells (SCs), Leydig cells (LCs), and peritubular myoid cells (PTMs). Traditionally, the testis has been regarded as an immunologically privileged and microbiologically sterile site due to its unique anatomical features.^11^^,^^12^^,^^13^ PTM surrounds the seminiferous epithelium to form a primary barrier, while the blood-testis barrier (BTB), located within the seminiferous tubules and formed by tight junctions between adjacent SCs, serves as a more effective barrier.^14^^,^^15^ This specialized anatomical arrangement safeguards germ cells from detrimental immune responses, as well as prevents the entry of molecules and bacteria from the blood vessels. Nevertheless, recent metagenome investigations have identified low-abundance bacteria within testicular tissue. Moreover, testicular BM dysbiosis has been linked to idiopathic non-obstructive azoospermia.^16^^,^^17^^,^^18^ However, the exact identity of these cell-associated bacteria and the specific host cell types with which they interact remain uncharacterized. Additionally, it is largely unknown at the molecular and genomic levels how the spatial distribution of testicular BM and the specific host-microbial cellular interactions impact the testicular immune microenvironment, function, and spermatogenic capacity.

Single-cell RNA sequencing (scRNA-seq) technologies have emerged as invaluable tools for unraveling cellular heterogeneity within mammalian tissues, including the testis. Multiple studies have utilized scRNA-seq technologies to characterize testicular cell types, elucidate developmental and age-related changes, and map the testicular microenvironment in infertile males.^19^^,^^20^^,^^21^^,^^22^ Recently, an improved scRNA-seq technology called invasion-adhesion-directed expression sequencing (INVADE-seq) was developed. INVADE-seq enhances the standard 10× Genomics 5′ scRNA-seq method by incorporating a bacterial 16S rRNA gene-targeting primer.^23^^,^^24^ This innovative approach has been successfully implemented to investigate tissue-associated microbiota at single-cell resolution in human cell lines and oral squamous cell carcinoma tumor tissue.^25^^,^^26^^,^^27^ Compared to traditional 16S RNA sequencing and scRNA-seq technologies, INVADE-seq enables detailed characterization of testicular BM composition, identification of bacteria-host interaction, and insights into the impact of testicular BM on reproductive capacity.

Previous histological and scRNA-seq studies in human and mouse have demonstrated that aging exerts diverse impacts on male fertility, including declining sperm quality, impaired BTB integrity, defective DNA repair in spermatogonia stem cells, and inflammation in multiple cell types.^19^^,^^20^^,^^21^^,^^22^^,^^28^^,^^29^ Aging also influences the human microbiota. From infancy to old age, microbiome features such as diversity, composition, and function of the microbiota in the skin, oral, and gut are under succession.^30^^,^^31^ However, the effects of aging on the testicular BM remain unknown. Given the reciprocal interaction between host and microbes, it is plausible that testicular aging may influence the testicular BM, and conversely, alterations in the testicular BM with aging could contribute to testicular changes. For instance, impaired BTB integrity could both result from and contribute to changes in the testicular BM during aging.^32^

To address these gaps, we utilized INVADE-seq to map cellular and molecular interactions between host cells and bacteria within the testis in over 70,000 single cells of young and old mice. We characterized the composition and distribution of the testicular BM and examined transcriptional and signaling changes in bacteria-associated cells across major testicular cell types, revealing a set of molecular mechanisms underlying testis-bacteria interaction. Collectively, our study not only provides novel insights into understanding of the testicular BM, its impact on reproductive capacity, and its relationship with aging but also serves as a valuable resource for the discovery of diagnostic biomarkers and therapeutic targets for BM- and age-associated male subfertility.

## Results

### Construction of single-cell transcriptomic atlas of young and older mice

**Figure 1:**
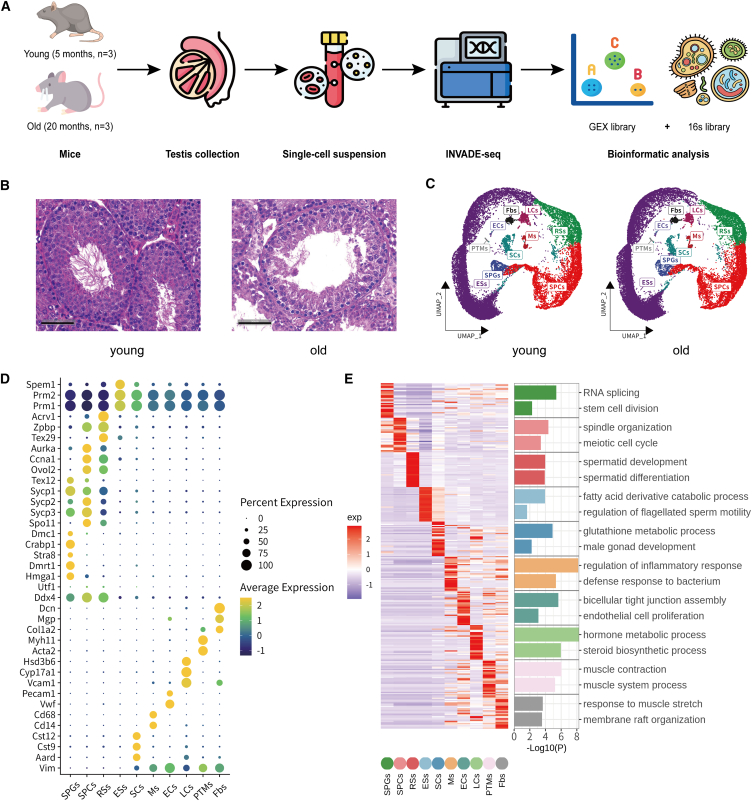
Construction of single-cell transcriptomic atlas of young and older mice testes (A) Schematic of the experimental design of this study. (B) H&E-stained testis sections from young and old mice, highlighting histological changes associated with aging. Scale bars represent 50 μm. (C) UMAP plots of the annotated testicular cell types from both young and old mice. Different colors represent distinct cell subsets. (D) Dot plot of marker gene expression across testicular cell types. Dot size indicates the percentage of expressing cells, and color intensity reflects average expression levels. Data are pooled from both young and old mouse samples. (E) Left: clustered heatmap of the top 30 differentially expressed genes in each cell cluster. The scaled gene expression levels are colored according to *Z* score. Right: the corresponding top 2 enriched biological processes in different cell clusters, and bar length corresponds to −log10(*p*), denoting statistical significance of pathway enrichment. Data are pooled from both young and old mouse samples.

To characterize the distribution of the testicular BM and specific host-microbial cellular interactions, we obtained whole testes of male mice aged 5 months (termed “young” mice, *n* = 3) and aged 20 months (termed “old” mice, *n* = 3). We performed INVADE-seq on all 6 samples using the 10× Genomics Chromium platform (Figure 1A). Histological examination revealed that some seminiferous tubules in aged mouse testes exhibited increased wall thickness and reduced germ cell layers, whereas most tubules maintained normal morphology (Figure 1B). This qualitative observation is consistent with previous reports describing localized degenerative changes in aged testes.^29^^,^^33^ Meanwhile, the sperm count in aging mice did not show a significant decrease, which is consistent with previous reports^20^^,^^29^ (Figure S1A).

A total of 72,326 single cells (33,738 from young mice and 38,588 from old mice) passed stringent quality control filtering and were included in the subsequent analysis (Figure S1A). Quality control criteria excluded cells with low gene counts (<200 genes), high mitochondrial gene percentage (>10%), or potential doublets, thereby ensuring data robustness. Dimensionality reduction was performed using principal-component analysis, and the top 50 principal components were applied for uniform manifold approximation and projection (UMAP) visualization. Clustering was conducted using the Louvain algorithm implemented in Seurat v.4.0. This analysis resolved 10 distinct cellular populations within the mouse testis, which were consistently detected in both young and old groups. Annotation of these clusters was based on the expression of canonical marker genes reported in prior single-cell studies of testis biology.^20^^,^^34^ Specifically, six major somatic cell populations were identified: SCs (*Aard*^+^), macrophages (*Cd68*^+^), endothelial cells (ECs, *Vwf*^+^), LCs (*Cyp17a1*^+^), PTMs (*Acta2*^+^), and fibroblasts (Fbs, *Dcn*^+^). In parallel, four germline populations representing successive stages of spermatogenesis were captured: spermatogonia (SPGs, *Crabp1*^+^), spermatocytes (SPCs, *Sycp3*^+^), round spermatids (RSs, *Acrv1*^+^), and elongating spermatids (ESs, *Prm1*^+^) (Figures 1C, 1D, and S1B). To further validate cluster identity, we conducted Gene Ontology (GO) enrichment analysis of the top 30 marker genes for each cluster. This analysis revealed pathways strongly associated with the specialized functions of the corresponding cell types, for example, steroid biosynthesis in LCs, tight junction organization in SCs, extracellular matrix organization in PTMs, and meiotic processes in SPCs (Figure 1E). These results confirm that our dataset robustly recapitulates the known cellular heterogeneity of the mouse testis while providing a comprehensive reference map at single-cell resolution.

### Aging-related cellular and transcriptional alterations

**Figure 2:**
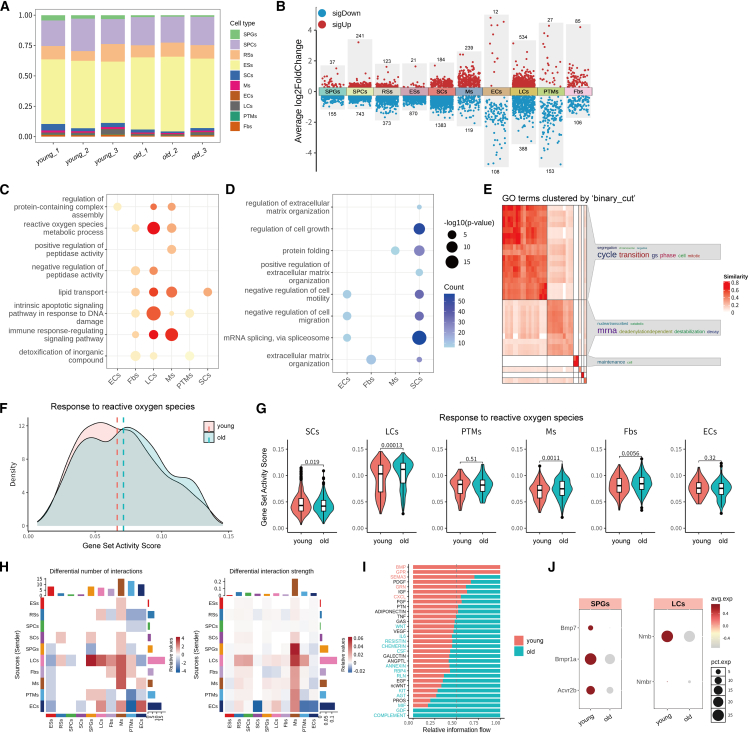
Single-cell analysis reveals functional and cellular interactions dynamics in testes during aging (A) Bar plot showing the relative proportions of distinct cell types (e.g., SPGs, SPCs, LCs, etc.) across different aging stages (young to old). Each color represents a specific cell type. (B) Quantitative comparison of the number of differentially expressed genes (DEGs) between young and aged testes across major testicular cell types. (C and D) Bubble plots showing representative GO terms enriched in upregulated (C) and downregulated (D) genes during testicular aging. Analyses were performed separately for each cell type, and the terms displayed represent those recurrently enriched across two or more testicular cell types. Bubble size indicates the number of genes involved in a pathway, and color intensity reflects statistical significance (–log10 *p* value). (E) Heatmap displaying clustered and merged Gene Ontology (GO) terms of downregulated genes in aged spermatogonia. The axis represents individual GO terms clustered by functional similarity. The *simplifyEnrichment* package was used to cluster and visualize functional enrichment results.^35^ (F and G) Density plots illustrating the distribution of gene set activity scores for the pathway “response to reactive oxygen species.” (F) shows the overall distribution across all testicular cells from young (red) and old (blue) mice. (G) shows the distribution stratified by individual cell types. The *y* axis represents the AUCell-derived gene set activity score, which reflects the enrichment level of the pathway in each single cell. (H) Heatmaps showing age-dependent changes in cell-cell communication, quantified by the number of predicted interactions (left) and the interaction strength (right) between testicular cell types. Results were calculated using the *CellChat* package, comparing young vs. old mice. (I) Bar graph depicting the relative information flow of ligand-receptor interactions between testicular cell types, illustrating representative information flow in testes of young (red) and old (blue). (J) Dot plots showing expression of BMP and GPR signaling genes inferred by CellChat.

We next investigated the testicular cellular composition changes during aging. All samples contained germ cells across developmental stages, but aged mice exhibited a significant reduction in spermatogonia proportion (Figures 2A, S2A, and S2B), indicating declining germline stem cell reserves. Meanwhile, we found that the transcriptional landscape of various testicular cell types underwent substantial remodeling during aging. Differentially expressed genes (DEGs) are provided in Table S1. Among testicular somatic cells, LCs and macrophages exhibited the most pronounced transcriptomic divergence, characterized by a larger number of DEGs and greater fold changes, particularly with more genes upregulated in aged testes (Figure 2B). This trend became even more evident when applying a stricter cutoff (fold change >1.5), as shown in Figure S2C. In addition, SCs also displayed a considerable number of DEGs, predominantly showing downregulation in aged mice, highlighting their susceptibility to age-related functional decline. These observations are consistent with the central roles of LCs and macrophages in maintaining testicular homeostasis and immune regulation. Differential expression and functional enrichment analyses were conducted for each annotated testicular cell type. GO enrichment of age-associated DEGs revealed that upregulated genes in old testes were enriched in pathways related to oxidative stress, protein synthesis, and immune signaling, whereas downregulated genes clustered in processes related to cell proliferation, migration, and extracellular matrix organization (Figures 2C and 2D), highlighting a systemic shift toward catabolic and inflammatory states alongside reduced anabolic and structural functions in aged testes. Notably, in spermatogonia, aging was associated with transcriptional repression of mitotic phase transition (G1/S phase) pathways (Figures 2E and S2D). Further analysis showed that in aged mice, the overall gene set score for “DNA damage response” was reduced in multiple germ cells (Figure S2E). Correspondingly, transcriptional downregulation of mitotic-associated genes (e.g., *Cdc6*, *Ccnd1*, and *Cdk1*) was observed in aged spermatogonia (Figure S2F). These results suggest a possible reduction in the self-renewal capacity of spermatogonia during aging.^36^^,^^37^ In addition, we observed that in aged mice, the scores of gene sets related to aging, such as the reactive oxygen species response pathway and the inflammatory response pathway, were increased, especially in LCs and macrophages (Figures 2F, 2G, and S2G). Furthermore, CellChat analysis revealed age-dependent rewiring of soma-germline interactions (Figure 2H), where bone morphogenetic protein (BMP) signaling (essential for spermatogonia self-renewal^38^^,^^39^^,^^40^) and G protein-coupled receptor (GPR) signaling (driving LC proliferation^41^) showed significantly weakened ligand-receptor interactions in aged testes (Figure 2I), manifested as reduced expression of genes *Bmp7* and *Nmb*, respectively (Figure 2J). Conversely, immune/inflammatory pathways (macrophage migration inhibitory factor (MIF), growth differentiation factor (GDF), and COMPLEMENT) were robustly activated (Figure 2I), exacerbating testicular microenvironmental inflammation.^42^ Collectively, our INVADE-seq data indicate that both testicular somatic and germ cells undergo significant molecular reprogramming during aging, which is largely consistent with previous findings.^19^^,^^20^^,^^29^

### Detection of low-abundance BM across various testicular cell types

**Figure 3:**
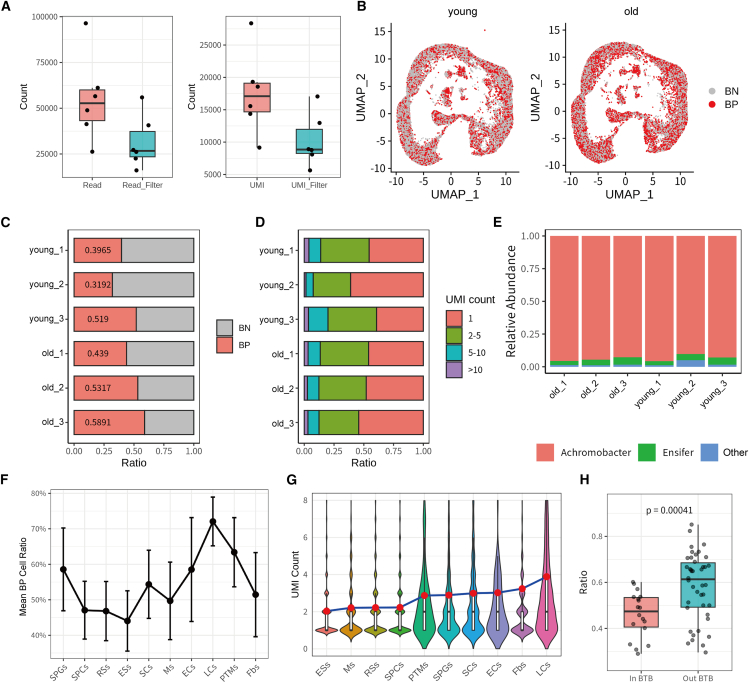
Integrated single-cell and microbial analysis confirmed microbial presence in the testicular microenvironment and demonstrated significant variation in microbial abundance among different testicular cell populations (A) Boxplots depicting the distribution of bacteria-associated sequencing metrics, including total reads, filtered reads (left), total UMIs, and filtered UMIs (right). (B) UMAP plots illustrating the single-cell transcriptomic landscape of testicular cells from young (left) and old (right) samples. Cells are colored by two states, BN (bacterial-negative, gray) and BP (bacterial-positive, red). (C) Bar plot showing the ratio of BN and BP cellular states for each sub-sample. (D) Bar plot representing the distribution of filtered bacteria UMI counts categorized into different ranges for sub-samples. (E) Bar plot displaying the relative abundance of microbial genus across all samples. (F) Line graph with error bars shows the mean ratio of bacterial-positive cells across different testicular cell types. Data are pooled from six biological replicates (*n* = 3 young, *n* = 3 old mice, data are represented as mean ± SEM, ∗*p* < 0.05, ∗∗*p* < 0.01 by Student’s *t* test). (G) Violin plots revealing the distribution of bacteria UMI counts per bacterial-positive cell for various testicular cell types. Data are pooled from six biological replicates (*n* = 3 young, *n* = 3 old mice, animals per group, mean ± SD,∗*p* < 0.05, ∗∗*p* < 0.01 by two-tailed *t* test). (H) Dot plot comparing the ratio of bacterial-positive cells between “In BTB” and “Out BTB” groups. The vertical axis represents the proportion of bacterial-positive cells, calculated as the number of bacteria-positive cells divided by the total number of cells in each group. Each dot corresponds to one specific testicular cell type for sub-samples, and the boxes show the median and interquartile range. “Out BTB” cell types including SCs, ECs, LCs, Fbs, PTMs, Ms, and SPGs, and “In BTB” cell types including SPCs, RSs, and ESs.

In the 16S library, an average of approximately 50,000 reads and 17,000 bacterial UMIs corresponding to bacterial sequences were detected per mouse. After filtering out non-target reads mapped to cells lacking corresponding identification in the gene expression library, an average of approximately 25,000 high-confidence reads remained per mouse. These findings demonstrate the robust performance of the INVADE-seq 16S bacterial enrichment strategy in detecting microbial presence (Figure 3A).

To investigate the distribution of bacterial UMIs across different testicular cell types, we performed UMAP projection for visualization. The results revealed a widespread presence of bacteria across various cell types (Figures 3B and S3A). Next, we quantified the proportion of bacterial-positive cells (bacterial UMI count ≥1) across different samples and found that roughly 50% of cells in each sample were bacterial positive (Figure 3C). These observations suggest that bacteria may either display a broad tropism toward various host cells or utilize specific mechanisms to migrate across different cell types. Our extended analysis focused on evaluating the bacterial transcriptional load within bacterial-positive cells. This examination revealed an average of approximately two microbial UMIs per cell, with nearly half of these cells harboring a single bacterial UMI (Figure 3D). These observations of minimal bacterial transcriptional activity suggest that bacterial biomass within the testis is scarce.

We further annotated the testicular bacterial composition and found that, at the genus level, *Achromobacter* was the most abundant, which can cause a broad range of infections in hosts, followed by *Ensifer* (Figure 3E). This unique distribution pattern dominated by a single bacterial genus may result from the specific targeting capability of INVADE-seq, which focuses on bacteria that adhere to or invade host cells. Given that adherent or intracellular bacteria have a heightened potential to directly regulate the physiological activities of their interacting cells,^43^^,^^44^ we proceeded with in-depth functional analysis to further elucidate these interactions.

### Heterogeneity of bacterial distribution within the testicular microenvironment

To investigate the potential spatial distribution and cell-type heterogeneity of bacterial distribution within the testicular microenvironment, we compared the bacterial communities of different testicular cell types. Our results revealed that there was no difference in the dominant bacterial types among various testicular cell populations (Figure S3B). However, the bacterial abundance exhibited heterogeneity across different cell groups (Figures 3F and 3G). Specifically, somatic cells, including SCs, ECs, LCs, Fbs, PTMs, and macrophages, along with spermatogonia, showed relatively higher bacterial abundances compared to spermatogenic cells (SPCs, RSs, and ESs). In these cell groups, we detected an elevated proportion of bacterial-positive cells, and the average UMI counts of microorganisms in bacterial-positive cells were also increased. This trend was consistently observed across all six biological replicates (Figure S3C).

Based on an analysis of the testicular physiological structure,^14^^,^^15^ we hypothesized that the higher bacterial abundance in SCs, ECs, LCs, Fbs, PTMs, and macrophages, along with spermatogonia, could be ascribed to their location outside the BTB. This anatomical positioning renders them more vulnerable to microbial exposure from the bloodstream. To validate this hypothesis, we conducted sample-paired differential analyses to compare the bacterial abundance between cells located inside and outside the BTB. The results indicated a significantly higher bacterial abundance in the cell groups located outside the BTB (Figure 3H). These results support our hypothesis that spatial localization relative to the BTB is a crucial determinant in shaping the bacterial distribution within the testicular microenvironment.

### Testicular bacterial abundance was elevated in aged mice and correlates with impaired BTB function

**Figure 4:**
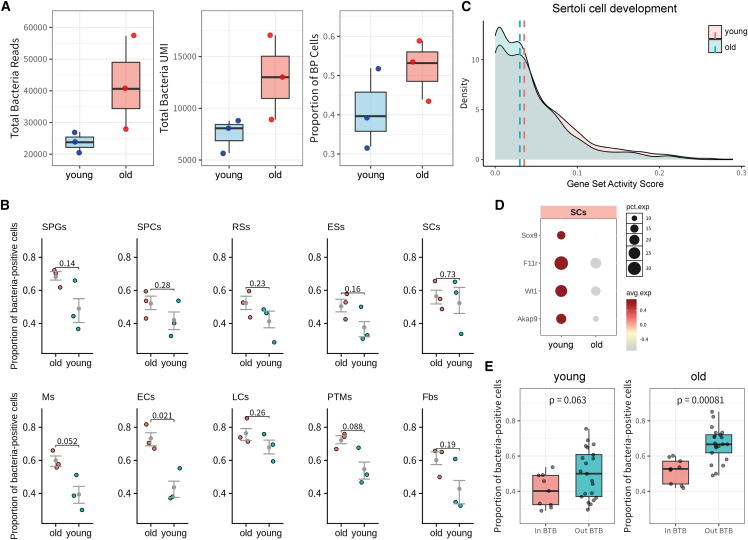
Age-related elevation of testicular bacterial abundance and its association with impaired BTB function (A) Boxplots depicting multiple metrics of testicular bacterial abundance in young and old mice, including total bacteria reads (left), total bacteria UMIs (middle), and the proportion of bacterial-positive (BP) cells (right). (B) Scatterplots comparing the bacterial abundance in various testicular cells from young and old mice. (C) Density plots visualizing the distribution of gene set score associated with “sertoli cell development” in aged sertoli cells (old, blue) and young sertoli cells (young, red). (D) Dot plots displaying the expression of key genes critical for sertoli cell development and tight junction formation of young and old samples. (E) Boxplot comparing bacterial-positive cell ratios between young and old mice for cell populations inside versus outside the BTB. The vertical axis denotes the proportion of bacterial-positive cells, calculated relative to the total cell count per population. Each dot represents one testicular cell type.

Previous studies, including our own findings, have shown that aging impacts the testicular microenvironment, leading to transcriptional alterations and multiple dysregulated biological processes in specific cell types. We hypothesized that the testicular BM landscape might also undergo changes during aging. Therefore, we compared the composition and abundance of testicular BM between young and aged mice. The results showed that aging did not alter the composition of the testicular BM (Figure 3E). However, we observed an upward trend in multiple metrics associated with bacterial abundance in aged mice (Figure 4A). Specifically, both the total UMI counts and sequencing read numbers, as well as the proportion of bacterial-positive cells and the bacterial UMI per positive cell, showed increasing tendencies in the aged group. However, these trends did not reach statistical significance, likely due to limitations such as the relatively small sample size and inherent intragroup variability. Nevertheless, the consistency of these trends across multiple metrics suggests a potential association between aging and elevated bacterial abundance in the testicular microenvironment. To further explore this observation, we performed a cell-type-specific analysis of bacterial abundance across distinct testicular cell populations. We found that all types of testicular cells exhibited a trend of increased bacterial abundance in old mice (Figure 4B). Notably, this tendency was most pronounced in specific cell types, including endothelial cells and macrophages (Figure S3D).

Our previous findings suggested that cell-type heterogeneity of bacterial distribution within the testicular microenvironment may be associated with the BTB. To investigate whether age-related changes in testicular BM are linked to BTB dysfunction, we performed a comparative analysis of DEGs in SCs from young and aged mice and found the overall gene set score for “sertoli cell development” was reduced in aged SCs but not in other testicular cells (Figures 4C and S3E). Specifically, genes critical for SC development and tight junction formation, such as *Sox9*, *F11r*, *Wt1*, and *Akap9*, were significantly downregulated in aged SCs (Figure 4D). These findings suggest that aging may impair the structural and functional integrity of the BTB, potentially contributing to increased bacterial abundance within the testicular microenvironment. In addition, we found that in aged mice, the difference in bacterial abundance between testicular cell populations inside versus outside the BTB became more pronounced (Figure 4E). While the young group showed only a modest trend (*p* = 0.063), the old group exhibited a statistically significant difference (*p* = 0.00081), with cells located outside the BTB carrying a higher bacterial load. This suggests that aging may impair the testis’s ability to eliminate bacteria, thereby exacerbating the disparity in bacterial abundance across the BTB.

### Testicular bacteria induce heterogeneous transcriptional alterations across testicular cell types and promote intercellular communication

**Figure 5:**
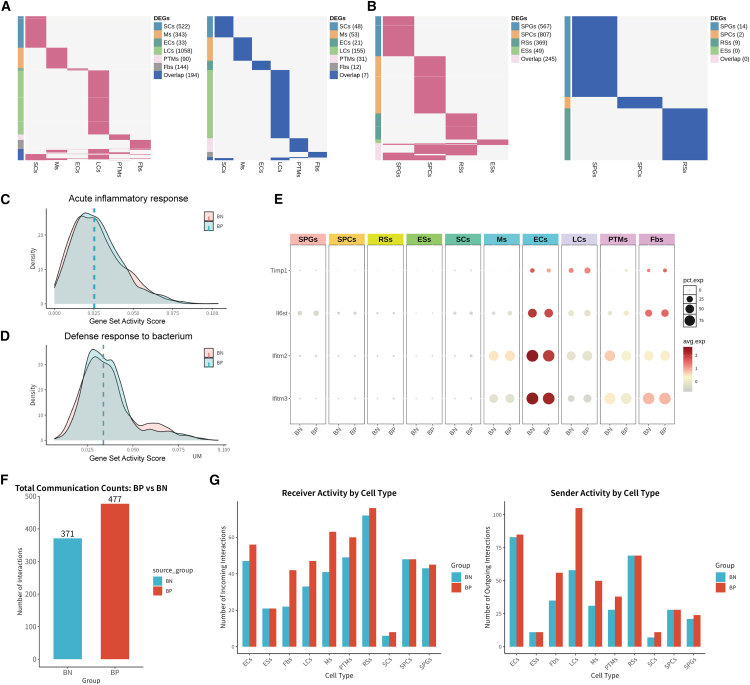
The transcriptional and cellular communication responses of testicular cells to bacterial adhesion/invasion (A) Heatmap displaying cell-type-specific and shared upregulated (left) and downregulated (right) DEGs between bacterial-positive (BP) and bacterial-negative (BN) states in testicular somatic cell types. (B) Heatmap displaying cell-type-specific and shared upregulated (left) and downregulated (right) DEGs between BP and BN states in testicular germ cell types. (C and D) Density plots visualizing the distribution of gene set score associated with “Acute inflammatory response” (C) and “Defense response to bacterium” (D) in BP (blue) and BN (red) cells. (E) Dot plots showing expression of key inflammation-associated genes across testicular cell types. (F and G) Bar plots quantify cellular communication dynamics. Total communication counts of BP (red) and BN (blue) cells (F). Detailed cell-type-specific changes in signal reception (G, left) and sending (G, right).

To investigate the impact of adherent or invasive bacteria on their interacting testicular cells, we identified DEGs in each testicular cell type (Figures 5A and 5B; Table S2). In bacterial-positive cells, the number of upregulated genes was significantly higher than that of downregulated genes (upregulated: 2,384 and 2,037 in somatic cells and germ cells, respectively; downregulated: 2,037 and 25 in somatic cells and germ cells, respectively). The majority of DEGs were cell-type specific, with LCs showing the highest number of DEGs. A small fraction of genes was shared across two or more somatic cell types, but functional enrichment analysis of these shared genes did not reveal any biologically meaningful pathways. Given that bacterial adhesion or invasion might trigger an inflammatory response, we focused on several inflammation-related pathways (e.g., the acute inflammatory response and the defense response to bacterium pathway). However, we observed no significant differences in the expression of those gene sets between bacterial-positive and bacterial-negative cells (Figures 5C and 5D). Correspondingly, key genes related to inflammation, such as *Timp1*, *Il6st*, and *Ifitm2/3*, showed comparable expression levels in both groups (Figure 5E). This finding suggests that while bacterial adhesion or invasion induces a robust transcriptional response in testicular somatic cells, this response is largely cell-type specific and does not appear to involve significant activation of canonical inflammatory pathways under the tested conditions. The absence of differential expression in inflammation-related genes may indicate that these cells employ alternative mechanisms to manage bacterial interactions without triggering overt inflammation.

To investigate whether bacteria invading or adhering to the cell surface alter the interactions of ligand-receptor between cell populations, we compared the communication networks of bacterial-positive and bacterial-negative testicular cells. Qualitatively, no differences in signaling networks were detected (Figures S4A and S4B). However, bacterial-positive cells exhibited a significant increase in communication quantity, with both signal sending and receiving enhanced, and particularly in testicular somatic cells (Figures 5F and 5G). These results suggest that bacteria adhering to the cell surface may act as intermediaries for intercellular communication.

### BM induces subtle transcriptional and functional changes in testicular somatic cells and spermatogenic cells

**Figure 6:**
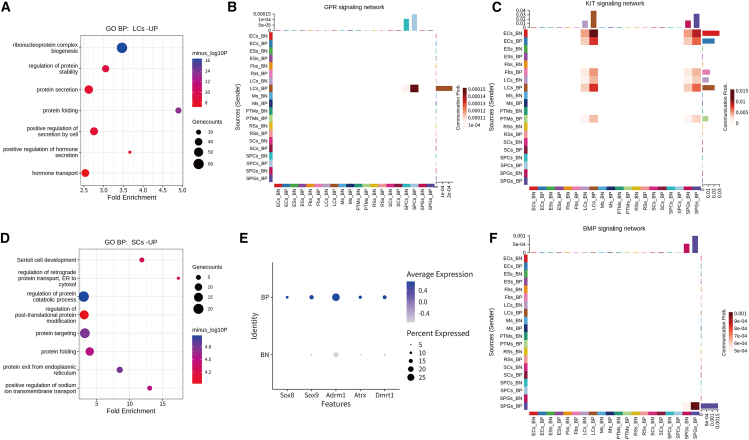
Cell-type-specific transcriptional and functional adaptations of testicular cells in response to bacterial adhesion/invasion (A) Representative GO terms of upregulated genes in bacterial-positive Leydig cells (LCs-UP). (B and C) Heatmap (by CellChat analysis) depicting the GPR signaling network (B) and KIT signaling network (C) in testicular cells. (D) Representative GO terms of upregulated genes in bacterial-positive sertoli cells (SCs-UP). (E) Dot plots showing expression of key genes associated with sertoli cell development and function in bacterial-positive and bacterial-negative cells. (F) Heatmap (by CellChat analysis) depicting the BMP signaling network in testicular cells.

We performed a focused analysis of LCs initially, as their transcriptional alterations were the most pronounced. GO enrichment analysis revealed that genes upregulated in bacterial-positive cells were significantly enriched in pathways related to ribonucleoprotein complex biogenesis, regulation of protein stability, protein folding, and secretion. Interestingly, the upregulated genes were enriched in the hormone synthesis and secretion pathway, which aligns with the specialized function of LCs (Figure 6A). Furthermore, AUCell analysis confirmed the activation of the steroid hormone biosynthesis pathway. Notably, this enrichment was distinctly absent among differential genes from other somatic cell types, underscoring the cell-type specificity of these transcriptional changes (Figures S5A and S5B). In particular, LCs displayed higher interaction numbers and interaction strength in bacterial-positive cells compared with bacterial-negative cells. For example, the GPR signaling network was activated characterized by the upregulation of *Nmb* and *Nmbr* genes in bacterial-positive LCs, which were associated with testosterone secretion (Figures 6B and S5C). Furthermore, the KIT signaling pathway increased between other testicular somatic cells and LCs, a pathway also known to play a key role in regulating self-renewal and senescence of LCs^45^^,^^46^ (Figures 6C and S5C). Collectively, our findings suggest that bacterial presence is associated with activation of testosterone-related transcriptional pathways in LCs.

While LCs exhibited the most robust responses to bacterial colonization, systematic profiling revealed subtle yet functionally relevant transcriptional adaptations across other testicular cell populations. In SCs, GO enrichment analysis identified significant upregulation of pathways critical for SC function, including support cell development, protein synthesis and transport, and cell-cell junction organization (Figure 6D). Regulated genes were upregulated in bacterial-positive SCs, including *Sox9*, *Adrm1*, and *Dmrt1* (Figure 6E). Suggesting bacteria may reinforce SC identity and protein homeostasis, thereby supporting their role in maintaining BTB integrity. Notably, quantitative bacterial mapping identified higher microbial loads in spermatogonia than in more advanced germ cells (Figures 3F and 3G), and the ligand-receptor interaction network activity in bacterial-positive spermatogonia was enhanced, particularly within self-renewal pathways: (1) potentiated SCF-KIT axis, a known mediator of stress-resilient spermatogonia stem cell maintenance, and (2) BMP signaling activation balancing stem cell quiescence versus differentiation^47^ (Figures 6C and 6F). These changes suggest transcriptional features in bacterial-positive spermatogonia that are compatible with a potential shift toward enhanced proliferative activity, possibly as an adaptive response to microenvironmental stress.

### BM promotes M2 polarization and enhances endocytosis/autophagy in testicular macrophages

**Figure 7:**
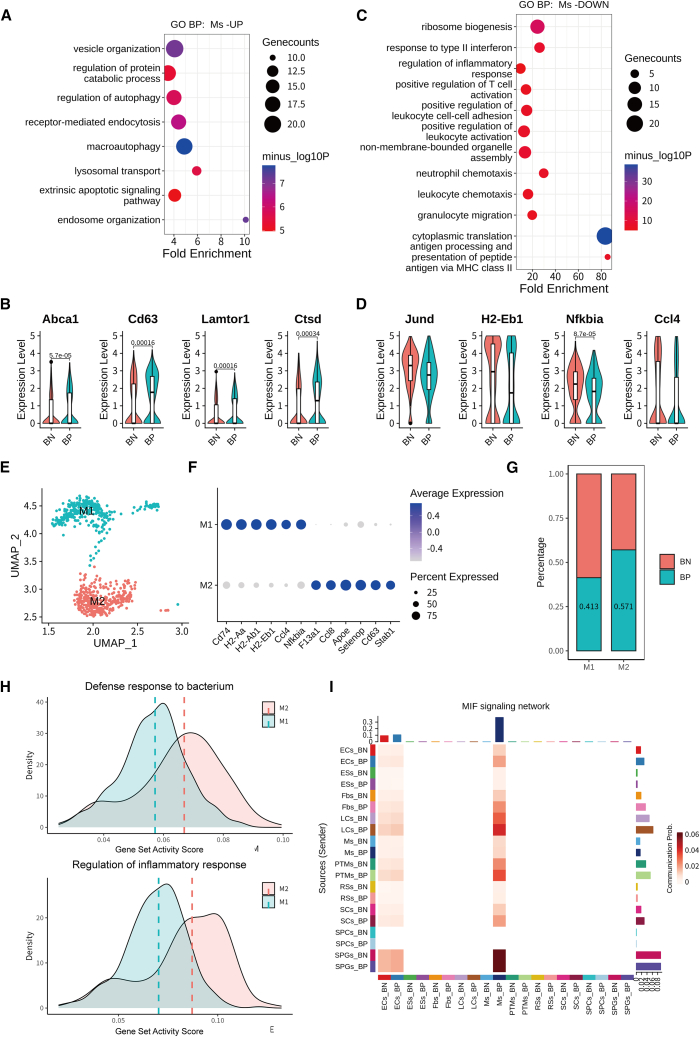
Transcriptional and functional reprogramming of testicular macrophages in response to bacterial interactions (A and B) Representative GO terms of upregulated genes in bacterial-positive macrophages (Ms-UP) (A) and dot plots showing expression of representative genes (B). (C and D) Representative GO terms of downregulated genes in bacterial-positive macrophages (Ms-DOWN) (C) and dot plots showing expression of representative genes (D). (E) UMAP plot of re-clustered macrophages, identifying two subclusters: M1-like (blue) and M2-like (red). Subcluster distribution reflects polarization states. (F) Dot plot of marker genes for M1-like (subcluster 1) and M2-like (subcluster 2) macrophages. (G) Bar plots of bacterial positivity rates in M1-like vs. M2-like subclusters. Higher BP enrichment in M2-like cells (57.10% vs. 41.35%) suggests enhanced bacterial interaction capacity in M2-polarized macrophages. (H) Density plots visualizing the distribution of gene set score associated with “Defense response to bacterium” (top) and “Regulation of inflammatory response” (bottom) in M1-like (blue) and M2-like (red) cells. (I) Heatmap (by CellChat analysis) depicting the MIF signaling network in testicular cells.

Testicular macrophages serve as the primary immune cells in mammalian testes.^48^ We next investigated bacteria-associated transcriptional changes between bacterial-positive macrophages and bacterial-negative macrophages. Interestingly, GO analysis revealed that bacteria-associated upregulated DEGs were enriched in pathways related to multiple processes in endocytosis and autophagy, including endosome organization and regulation of autophagy, receptor-mediated endocytosis (Figure 7A). Transcriptional upregulation of endocytosis/autophagy-associated genes (e.g., *Abca1*, *Cd63*, *Lamtor1*, and *Uvrag*) was further observed in bacterial-positive macrophages (Figure 7B). Conversely, GO analysis of downregulated genes in bacterial-positive macrophages highlighted significant enrichment in pro-inflammatory pathways, including antigen processing/presentation via major histocompatibility complex class II, leukocyte chemotaxis, and positive regulation of T cell activation (Figure 7C), with key pro-inflammatory genes (e.g., *Jund*, *H2-Eb1*, *Nfkbia*, and *Ccl4*) showing transcriptional repression (Figure 7D).

To investigate the cellular basis of these transcriptional dichotomies, we performed re-clustering of macrophages, identifying two distinct subclusters (Figure 7E). Subcluster 1 exhibited a pro-inflammatory M1-like signature, marked by high expression of *Cd74*, *H2-Ab1*, and *Ccl4*. In contrast, subcluster 2 displayed canonical M2-like features, including upregulation of *Ccl8*, *Apoe*, and *Stab1* (Figure 7F). Notably, the transcriptomic profile of bacterial-positive macrophages strongly overlapped with subcluster 2 (M2-like), whereas bacterial-negative cells were more closely associated with subcluster 1 (M1-like). Quantification of bacterial positivity rates revealed a significant enrichment of bacterial-positive cells in the M2-like subcluster (57.10%) compared to the M1-like subcluster (41.35%) (Figure 7G), and the signature score of M2-like macrophages was relatively higher in bacterial-positive cells (Figure S6A). This suggests that M2-like macrophages may have an increased tendency to interact with bacteria, potentially mediated by transcriptional upregulation of endocytic/autophagic activity. In addition, comparative analysis revealed that the proportion and signature score of M2-like macrophages was relatively higher in aged testes (Figures S6B and S6C), consistent with the increased bacterial abundance observed in aged mice (Figures 4A and 4B). Furthermore, we observed that pathways including “Defense response to bacterium,” “Endocytosis,” and “Regulation of inflammatory response” were activated in M2-like macrophages compared to M1-like macrophages (Figure 7H). Meanwhile, signaling pathways related to the immune system, such as the MIF signaling pathway, were increased in bacterial-positive macrophages (Figure 7I). Taken together, our findings suggest that under low bacterial burden, bacterial-positive testicular macrophages are more frequently associated with an M2-like polarization state, which may promote non-inflammatory bacterial clearance via enhanced endocytosis and autophagy.^49^ Further validation with direct immunostaining or flow cytometry is required.

## Discussion

The mammalian testis has long been regarded as a relatively immune-privileged site due to the protective function of the BTB.^11^^,^^12^^,^^13^^,^^50^ However, our study challenges this traditional view by revealing the ubiquitous yet low-abundance presence of BM across various testicular cell types. Using single-cell resolution analysis, we provide the first comprehensive map of testicular BM composition, its cellular distribution patterns, age-related changes, and functional impacts on the testicular microenvironment. These findings not only expand our understanding of testicular immunology but also shed new light on potential mechanisms underlying BM- and age-associated male subfertility.

Our findings extend prior reports of microbial presence in the male reproductive tract. Early studies using bulk 16S rRNA or metagenomic sequencing identified low-abundance bacteria in semen and testicular tissues^16^^,^^17^ but were limited by tissue-level averaging and the potential for contamination. By leveraging INVADE-seq, we resolve the bacterial landscape at single-cell resolution, confirming that bacteria-associated transcripts exist in both somatic and germ cells and revealing marked cell-type heterogeneity in bacterial load. In particular, the enrichment of bacterial UMIs in somatic cells and early-stage germ cells outside the BTB is consistent with recent observations that aging-related microvascular leakage and immune activation predispose these cells to external exposure.^19^^,^^20^^,^^21^^,^^22^ Together with recent human scRNA-seq atlas, our dataset establishes a comparative framework for investigating host-microbe co-regulation at single-cell resolution.

The age-dependent elevation of bacterial abundance observed here parallels reports of BTB weakening and oxidative stress accumulation during testicular aging.^19^^,^^32^^,^^42^ Our data provide a potential link between these phenomena: dysregulation of SC junction genes (e.g., *Sox9*, *F11r*, and *Wt1*) and local microbial translocation may jointly drive chronic microinflammation. Similar microbiota-barrier interactions have been documented in gut and blood-brain interfaces where age-related leakage permits bacterial products to activate innate immune responses.^51^^,^^52^^,^^53^ These parallels suggest that the testis may undergo a similar age-associated permeability shift, linking microbial exposure to oxidative and inflammatory stress.

Functionally, the cell-type-specific responses we identified further contextualize previous bulk transcriptomic studies reporting LC senescence and macrophage polarization with age.^32^^,^^36^^,^^48^ Our single-cell data demonstrate that bacteria-associated LCs upregulate steroidogenic genes (*Nmb/Nmbr* axis), which may represent an initial compensatory mechanism to maintain reproductive function in the face of microbial challenge. However, chronic activation of this pathway could lead to oxidative stress and cellular damage, ultimately contributing to age-related decline in LC function. Meanwhile, macrophages in contact with bacteria exhibit enhanced autophagy and M2-like polarization, a state previously implicated in anti-inflammatory clearance but also in immune tolerance that permits pathogen persistence.^54^^,^^55^ Notably, bacterial-positive cells displayed a relative enrichment of intercellular signaling networks compared to bacterial-negative cells, suggesting that bacteria may act as modulators of intercellular crosstalk. These finding underscores the potential of bacteria to reshape the testicular microenvironment by modulating ligand-receptor interactions, which could either enhance tissue resilience or disrupt homeostatic balance.

The link between increased testicular BM load and age-related reproductive decline raises the possibility that microbial dysbiosis could serve as a novel diagnostic biomarker for male subfertility. Additionally, pharmacological targeting of specific microbial taxa or their molecular interactions with host cells may offer innovative therapeutic strategies. For example, modulating the M2 macrophage polarization or restoring BTB integrity could potentially mitigate the negative effects of microbial invasion on testicular function. However, further research is needed to establish causality and determine the feasibility of such interventions. In conclusion, our single-cell analysis provides a detailed portrait of the testicular microbiome and its dynamic changes during aging. These findings highlight the dynamic and bidirectional interplay between the microbiome and testicular cells, providing preliminary mechanistic insights into male reproductive health and suggesting potential targets for BM- and age-associated male subfertility.

### Limitations of the study

While our study provides valuable insights into the testicular microbiome, several limitations should be acknowledged. First, from a technical perspective, while INVADE-seq effectively captures cell-associated bacteria, it may underestimate low-abundance taxa or fail to distinguish closely related strains due to the inherent limitations of 16S rRNA gene sequencing. Additionally, our approach focused solely on adherent or invasive bacteria, neglecting free-living microorganisms in the interstitial fluid or lumen of seminiferous tubules.^8^^,^^9^^,^^56^ These free bacteria might secrete metabolites or activate immune cells, indirectly affecting testicular homeostasis. Integrating bulk metagenomics with single-cell data would be essential to comprehensively characterize both cell-associated and free-living microbiome fractions. Moreover, the bacterial-positive cells detected in our dataset contained only very sparse bacterial UMIs, making it technically challenging to separate bacterial-positive from bacterial-negative cells for targeted experimental validation. Consequently, direct confirmation of differential gene expression between bacterial-positive and bacterial-negative cells using RT-qPCR or *in situ* hybridization could not be achieved at this stage. Second, from a mechanistic perspective, although our study establishes an association between increased microbial load and impaired BTB function in aging testes, causal relationships remain unproven. Future research should employ functional validation experiments such as antibiotic treatment to deplete the microbiome and observe BTB restoration, or genetic models with BTB disruption to directly assess the impact of microbial leakage on reproductive capacity. Third, our findings are based on a murine model, and the composition, abundance, and host-microbiome interactions in human testes may differ significantly. Cross-species comparative studies using primate models or human testicular biopsies are essential.

## Resource availability

### Lead contact

Requests for further information and resources should be directed to and will be fulfilled by the lead contact, Jianteng Zhou (zjt13@xzhmu.edu.cn).

### Materials availability

This study did not generate new unique reagents. All resources and materials reported in this paper will be shared by the lead contact upon request.

### Data and code availability

• The INVADE-seq data generated in this paper were deposited in the GEO database with accession number GSE303193.
• This study does not report any original code.
• Any additional information required to reanalyze the data reported in this paper is available from the lead contact upon request.

## Acknowledgments

This work was supported by the 10.13039/501100001809National Natural Science Foundation of China (82402187), the 10.13039/501100004608Natural Science Foundation of Jiangsu Province (BK20241953), Xuzhou Science and Technology Innovation Project (KC23009), and Xuzhou Key Research and Development Program (KC22096). We are grateful to OE Biotech Co., Ltd. (Shanghai, China) for sequencing service. We are grateful to Ms. Huan Zhang for her language and editorial help.

## Author contributions

J.Z.: methodology, investigation, visualization, and writing – original draft; Y.L.: methodology, investigation, and visualization; T.Z.: investigation and visualization; K.Y.: methodology and investigation; C.Z.: methodology; R.Z.: investigation and visualization; X.Z.: methodology and investigation; D.Z.: investigation and visualization; X.D.: investigation and visualization; Y.Q.: writing – review and editing; C.H.: conceptualization and writing – review and editing; Z.Z.: conceptualization, supervision, funding acquisition, and writing – review and editing.

## Declaration of interests

The authors declare no competing interests.

## STAR★Methods

### Key resources table

**Table:** 

REAGENT or RESOURCE	SOURCE	IDENTIFIER
**Chemicals, peptides, and recombinant proteins**		

Collagenase type IV	GIBCO	Cat# 17104019
Ethanol (gradient, 70–100%)	Sinopharm Chemical Reagent Co.	N/A
Hematoxylin solution	Beyotime Biotechnology	Cat# C0105
Eosin Y solution	Beyotime Biotechnology	Cat# C0106
Neutral mounting medium	Solarbio	Cat# G8590

**Critical commercial assays**		

Chromium Next GEM Single Cell 5' Kit v2	10x Genomics	PN-1000263

**Deposited data**		

scRNA-seq data (raw data)	This paper	GSE303193

**Experimental models: Organisms/strains**		

Male C57BL/6J mice	Zhishan Institute of Health Medicine Co., Ltd (Beijing, China)	zsc5722m

**Software and algorithms**		

Seurat v4.0	Satija Lab	https://satijalab.org/seurat
Cell Ranger v6.1.2	10x Genomics	https://support.10xgenomics.com
Kraken2 v2.1.2	Wood Lab	https://ccb.jhu.edu/software/kraken2
ggplot2 v3.4.0	CRAN	https://cran.r-project.org
clusterProfiler	Bioconductor	https://bioconductor.org/packages/clusterProfiler
INVADEseq pipeline	Galeano et al., Nat Protoc, 2023	https://github.com/FredHutch/invadeseq
CellChat	Bioconductor	https://github.com/sqjin/CellChat
AUCell	Bioconductor	https://github.com/aertslab/AUCell
R v4.3.2	R v4.3.2	R v4.3.2

### Experimental models and study participant details

#### Mice

C57BL/6 male mice at 5 months (young) and 20 months (older) were used, with 3 biological replicates per group. Mice were purchased from Zhishan Institute of Health Medicine Co., Ltd (Beijing, China) and housed under specific pathogen-free conditions and *ad libitum* access to food and water. All animal experiments in this study were conducted in strict accordance with the guidelines of the Institutional Animal Care and Use Committee of Xuzhou Medical University and were approved by the Ethics Review Board No.202302T006. The welfare and handling of animals were prioritized to minimize suffering.

### Method details

#### Epididymal sperm count

The unilateral cauda epididymis from each mouse was dissected, minced into small pieces, and transferred into an Eppendorf tube containing 1 ml of 1× PBS (68.5 mM NaCl, 1.3 mM KCl, 5.0 mM Na2HPO4, 0.9 mM KH2PO4). The tissue suspension was incubated at 37°C for 30 min to allow sperm to be released into the medium. Subsequently, 10 μl of the sperm suspension (diluted 1:10 in 1× PBS) was loaded onto a hemocytometer, and sperm were counted under a light microscope. Each sample was analyzed in triplicate, and the average of the three replicates was used as the final sperm count value per mouse.

#### Preparation of single-cell suspension

Tissue dissociation was performed under sterile conditions. Freshly collected testes were washed twice with ice-cold RPMI 1640 medium containing 0.04% BSA, minced into ∼0.5 mm^3^ pieces with surgical scissors, and transferred to an enzymatic digestion solution containing RPMI 1640 (Conring, cat. no. 10-040-CVR), 0.04% BSA (MACS, cat. no. 1000076), and 0.2% collagenase II (Gibco, cat. no. 17101015). Samples were incubated at 37°C for 30–60 min with gentle inversion every 5–10 min. The resulting cell suspension was filtered through a 40-μm cell strainer, centrifuged at 300 × g for 5 min at 4°C, and the pellet was resuspended in medium. Red blood cells were lysed using Miltenyi buffer for 10 min at 4°C. After centrifugation, the pellet was washed once and resuspended in 1 ml RPMI 1640 with 0.04% BSA. Cell concentration and viability were assessed using a Luna-FL counter or Trypan Blue staining.

#### Single-cell transcriptome sequencing

Chromium Next GEM Single Cell 5' Kit v2 (10x Genomics, PN-1000263) was used for scRNA-seq and INVADE-seq according to the User Guide (CG000331). Single cell suspension was adjusted to 700-1200 cells/μL. 20,000 cells were mixed with reverse transcription Master Mix, 16S primer (5′-GGGTTGCGCTCGTTG-3′, final concentration 16 μmol/L) and loaded into a Chromium chip K with gel beads and partitioning oil to generate GEMs on a 10x Chromium Controller, following with reverse transcription PCR and preamplification of the full-length cDNA. The cDNA QC was performed using Agilent 4150 TapeStation (Agilent Technologies). The cDNA was used to construct the transcriptome sequencing library using a 10x Genomics Library Construction Kit (PN-1000190). High-throughput sequencing was performed on the Illumina NovaSeq X Plus sequencer with PE-150 mode.

#### INVADE-seq library sequencing

The INVADE-seq bacterial 16S rRNA library was constructed according to Galeano Niño et al.^26^ with minor modification.16S rRNA primers for amplification were Forward Primer (FP): 5′-GATCTACACTCTTTCCCTACACGACGCTCTTCCGATCT-3′ and Reverse Primer (RP):5′-GTGACTGGAGTTCAGACGTGTGCTCTTCCGATCTTCACGRCACGAGCTGACGAC-3′. The 16S rRNA gene was enriched from 2 μL of cDNA using 1 μmol/L each of FP and RP, and 50 μL of Amp Mix (10x Genomics, PN-2000047) with the following PCR program: initial denaturation at 98°C for 45 sec; 35 cycles of 98°C for 20 sec, 67°C for 30 sec (with a 2 C/sec gradient warming), and 72°C for 1 min; and a final extension at 72°C for 1 min. The amplified products were purified using 0.8x SPRI beads (Beckman) and size-selected on an agarose gel to recover fragments between 955-1215 bp. A second round of amplification was performed on the recovered products under the same conditions but with 20 cycles. After purification with 0.8x SPRI beads, the product size was confirmed using an Agilent 4150 TapeStation. Following the Chromium Next GEM Single Cell 5' Kit v2 (Dual Index) User Guide, 50 ng of the purified product was adjusted to 30 μL and mixed with 50 μL of Amp Mix (PN-2000047) and 20 μL of Dual Index TT Set A (PN-1000215) for PCR amplification: 98°C for 45 sec; 15 cycles of 98°C for 20 sec, 54°C for 30 sec, and 72°C for 20 sec; and a final extension at 72°C for 1 min. The amplified products were purified with 0.8x SPRI beads, size-selected for 955-1215 bp fragments on an agarose gel, and qualified using an Agilent 4150 TapeStation. High-throughput sequencing was performed on an Illumina NovaSeq X Plus sequencer in PE-150 mode.

#### scRNA-seq data processing

Raw FASTQ files were processed using Cell Ranger (v9.0.0, 10x Genomics) to align reads to the mm10 genome and generate unique molecular identifier (UMI) count matrices. Subsequent analysis was performed using Seurat^57^ (v4.0.0) in R. Low-quality cells and multiplets were filtered based on: (1) detected genes (<200), (2) total UMIs (<1,000), (3) log10GenesPerUMI (<0.7), (4) mitochondrial UMI percentage (>10%), and (5) hemoglobin UMI percentage (>5%). DoubletFinder^58^ (v2.0.3) was applied to identify potential doublets.

Normalized data were subjected to principal component analysis (PCA), and the first 50 principal components were used for uniform manifold approximation and projection (UMAP) visualization and clustering via the Louvain algorithm in Seurat. Cell types were allocated to each cluster using the abundance of known marker genes as described previously.

#### Generation of bacteria UMI matrices from the INVADE-seq bacterial 16S rRNA library

The bacterial 16S rRNA gene enrichment library was processed to generate UMI matrices according to previous reports^23^ using a workflow integrating CellRanger, BEDTools,^59^ Trimmomatic,^60^ Picard, and GATK PathSeq.^61^ Raw FASTQ files were first processed with CellRanger count to align reads to the reference genome. The resulting BAM file was converted to FASTQ format using BEDTools, and adapter sequences were trimmed from Read 1 using Trimmomatic with parameters to remove low-quality bases and clip the first 15 nucleotides. The trimmed FASTQ was converted back to BAM format with Picard FastqToSam and processed using GATK PathSeq to filter host reads and identify bacterial sequences, using specified reference databases for host and microbial genomes. The output BAM file containing bacterial annotations and summary CSV were then integrated with the corresponding CellRanger output from the gene expression (GEX) library using a custom Python script. This script associated bacterial 16S sequences with valid cell barcodes identified in the GEX library, generating files including genus-level assignments per cell, raw read mappings, and validated bacterial-cell associations.

#### Integration of bacteria UMI matrix with single-cell data

The bacterial UMI CSV table was imported as a dataframe with row names set to cell barcodes. The total bacterial UMIs per cell were calculated and added as a new column. This matrix was then merged with the Seurat object containing the single-cell transcriptomic data using *AddMetaData*, ensuring all cells without detected bacteria were assigned zero values. Cells were classified as bacterial-positive if they harbored ≥1 bacterial UMIs.

#### Identification of differentially expressed genes (DEGs) and GO enrichment analysis

To identify DEGs between Young and Older or bacterial-positive and bacterial-negative groups in each cell type, we use the Seurat FindMarker function based on normalized TPM expression values. The Genes with expression level differences greater than 1.2 and *p*-value < 0.01 were defined as DEGs. DEGs between Young and Older is provided in Table S1, and DEGs between bacterial-positive and bacterial-negative groups in each cell type is provided in Table S2. Gene Ontology (GO) enrichment analysis for biological processes was performed using clusterProfiler^62^ v4.2.2, with significance defined as *p* value < 0.01, and a gene count greater than 3 as significant pathways. Subsequently, the output of the clusterProfiler analysis was processed using simplifyEnrichment^35^ v1.14.0, with a similarity cutoff of 0.7 was applied to cluster and merge redundant GO terms.

#### Cell–cell communication analysis

Cell-cell communication analysis was conducted utilizing the CellChat^63^ v2.1.2. “CellChatDB.mouse” was set up as the ligand-receptor interaction database, cell-cell communication analysis was then performed via the default setting. The significance of communication links between cell clusters was evaluated using the *computeCommunProb* function, with a probability threshold of 0.01 set to filter out weak interactions. To visualize and quantify the communication network, CellChat generated several metrics, including communication probability matrices, network plots, and heatmaps.

#### AUCell

To quantify BM-associated transcriptional programs, we employed the AUCell (Area Under the Cell-activity curve) package (version 1.26.0) in R. AUCell scores individual cells for gene set enrichment (GSE). Gene sets were derived from MSigDB Hallmark gene sets, and cell scores were normalized across samples. We visualized the AUCell scores using density and violin plots to illustrate the distribution of gene set activity across different groups or cell clusters.

#### H&E staining

Testicular tissues from the young and older mice were fixed in 4% paraformaldehyde, dehydrated, and embedded in paraffin. H&E staining was performed using standard protocols: hematoxylin for 5 min, differentiation in acid alcohol, bluing in ammonia water, and eosin staining for 3 min. Slides were dehydrated, cleared, and coverslipped. Quantitative analysis of seminiferous tubule diameter and cell density was performed using ImageJ.

### Quantification and statistical analysis

All statistical analyses were performed using R (v4.3.2) unless otherwise specified. Statistical details for each analysis, including the statistical tests used, exact n values, definitions of n, measures of central tendency, dispersion metrics, and significance thresholds, are reported in the corresponding figure legends and results sections.

For comparisons between two groups (e.g., young vs. old; bacterial-positive vs. bacterial-negative cells), two-tailed unpaired Student’s t-tests were applied unless noted otherwise. For gene set–related analyses (e.g., AUCell, enrichments), statistical significance was determined using the built-in scoring procedures of the respective tools (AUCell, clusterProfiler, simplifyEnrichment, CellChat) with multiple-testing correction where applicable.

For single-cell analyses, n refers to the number of individual cells after quality control filtering. For bulk-level comparisons (e.g., sperm count, bacterial metrics per mouse), n refers to the number of biological replicates (3 young mice and 3 old mice). Measures of central tendency and variability are reported as mean ± standard deviation (SD) unless specified.

Differentially expressed genes (DEGs) were identified using Seurat’s FindMarkers function with the following thresholds: log fold-change > 1.2 and *p* value < 0.01. Gene Ontology enrichment was performed using clusterProfiler with significance defined as *p* < 0.01 and minimum gene count > 3. Cell-cell communication analyses were conducted using CellChat with a communication probability cutoff of 0.01 to filter weak interactions.

All statistical tests were two-sided, and *p* < 0.05 was considered statistically significant unless indicated otherwise. Exact values, significance indicators, and test types can be found in the figure legends.

## Supplemental information

Document S1. Figures S1–S6

Table S1. Differentially expressed genes between young and older mice

Table S2. Differentially expressed genes between bacteria-positive cells and bacteria-negative cells
