# Supplementary material for: Single-cell analysis of testicular bacterial microbiome changes during aging and effect on reproductive capacity in mice
Source: iScience. 2025 Nov 22;28(12):114174. doi: 10.1016/j.isci.2025.114174 (PMC12719757; doi:10.1016/j.isci.2025.114174)
Supplement: Document S1. Figures S1–S6 [file mmc1.pdf]

iScience, Volume *28*

Supplemental information

Single-cell analysis of testicular bacterial microbiome changes during aging and effect on reproductive capacity in mice

Jianteng Zhou, Ying Li, Tao Zhu, Kexin Yang, Cheng Zhang, Ruoqi Zhang, Xinrong Zhang, Dianshuang Zhou, Xiaoyue Ding, Yu Qiao, Conghui Han, and Zuobin Zhu

A

Mice ID	Age (Month)	Sperm Count (10^6)	Total cells after QC	Median genes per Cell
5A	5	18817	10084	1500
5B	5	26767	13442	1703
5C	5	20267	10212	2099
20A	20	11333	14184	1660
20B	20	21467	12839	1746
20C	20	30667	11565	1788

B

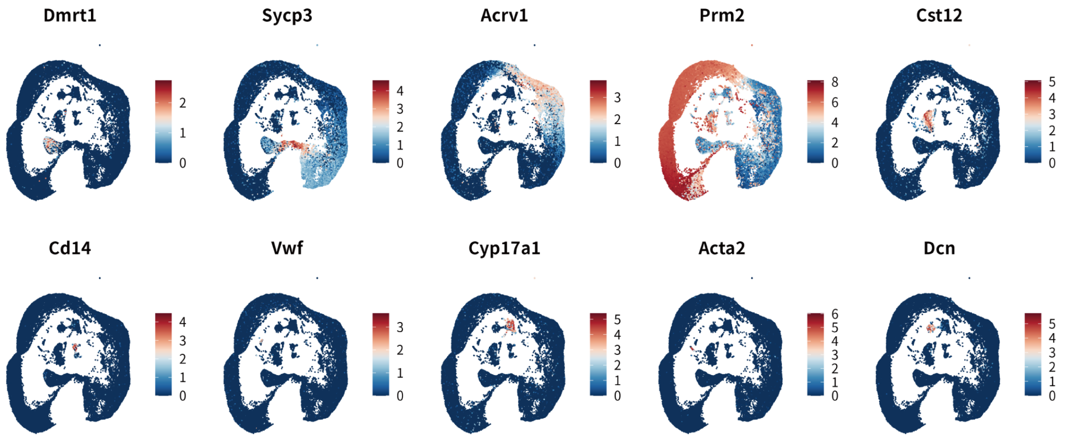

Figure S1. Information about mice and quality control of scRNA-seq

A) Summary table of experimental metadata for individual mice. Columns include: mouse ID, age (5 months = young; 20 months = old), sperm count, total cells after QC, and median genes per cell. Sperm counts are expressed as 10^6 spermatozoa per mouse, calculated from the cauda epididymis. "Total cells" and "Median genes per cell" refer to the testicular single-cell suspensions captured by the 10x Genomics platform.

B) Feature plots of cell-type–specific marker genes overlaid on the UMAP projection of testicular single cells. Scale bar indicates normalized log-transformed gene expression levels. Data shown were pooled from young (5 months) and old (20 months) mice.

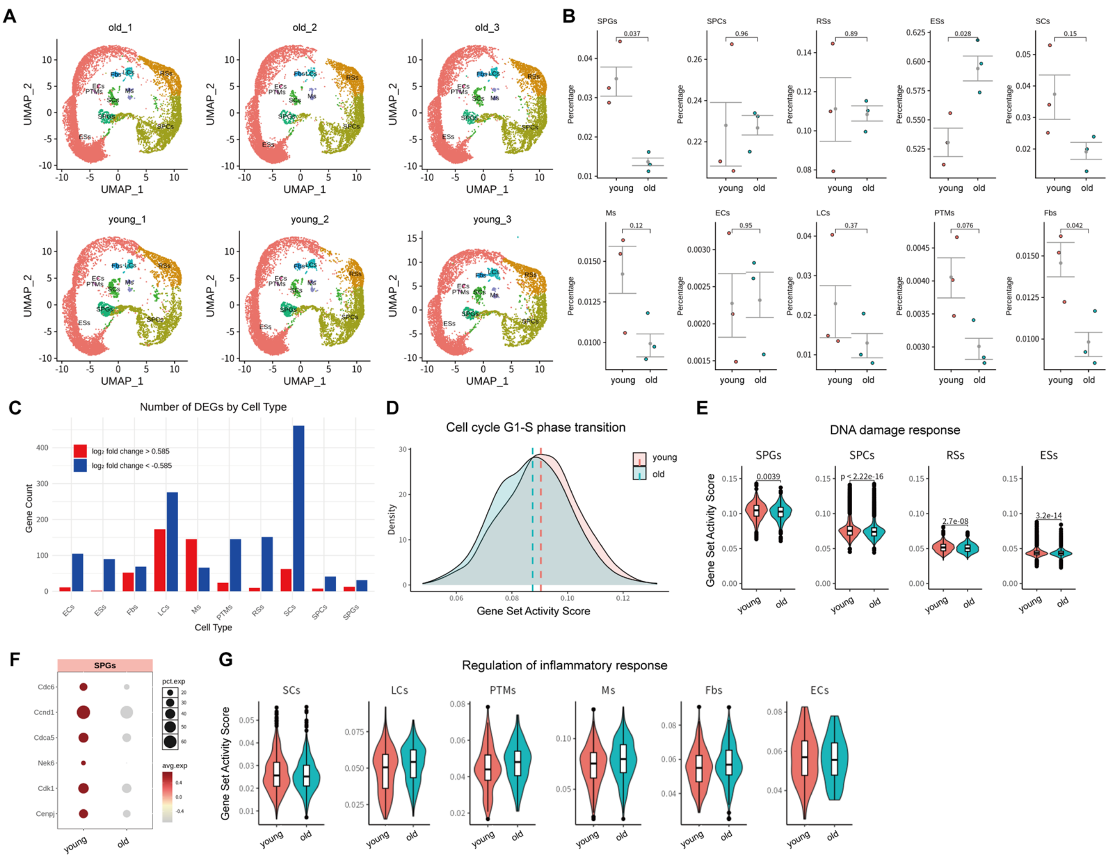

Figure S2. Cell transcriptomic analyses of testicular cells from young and old mice, highlighting age-associated changes in cell populations, functional pathways, and cellular states

A) UMAP plots of testicular cells for individual young and old samples. Each plot shows the distribution of different cell clusters (color-coded).

B) Dot plots comparing the proportion of various testicular cell types (SPGs, SPCs, RSs, ESs, Ms, ECs, LCs, PTMs, Fbs) between young and old samples. The y-axis represents cell type proportions, and the x-axis denotes sample groups (young and old). Statistical significance is indicated, revealing age-related changes in cell type distribution.

C) Quantitative comparison of DEGs between young and aged mouse testes across major testicular cell types under a stricter threshold. The number of DEGs (Y-axis) was calculated for each annotated cell type (X-axis) using Seurat with an absolute $\log_2$ fold change > 0.585 (expression level differences greater than 1.5). Red bars represent genes upregulated in aged testes, and blue bars represent genes downregulated in aged testes.

D) Density plots visualizing the distribution of gene set score associated with 'Cell cycle G1/S phase transition' in young (red) and old (blue) samples.

E) Violin plots of 'DNA damage response' gene set activity scores (AUCell) across different testicular cell types in young (red) and old (blue) mice. While global differences appear subtle, consistent downward shifts are observed in spermatogonia and spermatocytes.

F) Dot plots displaying the expression of representative genes of 'Cell cycle G1/S phase transition' pathway in SPGs from young and old samples.

G) Violin plots of gene set scores for 'regulation of inflammatory response' across different testicular cell types in young (red) and old (blue) samples.

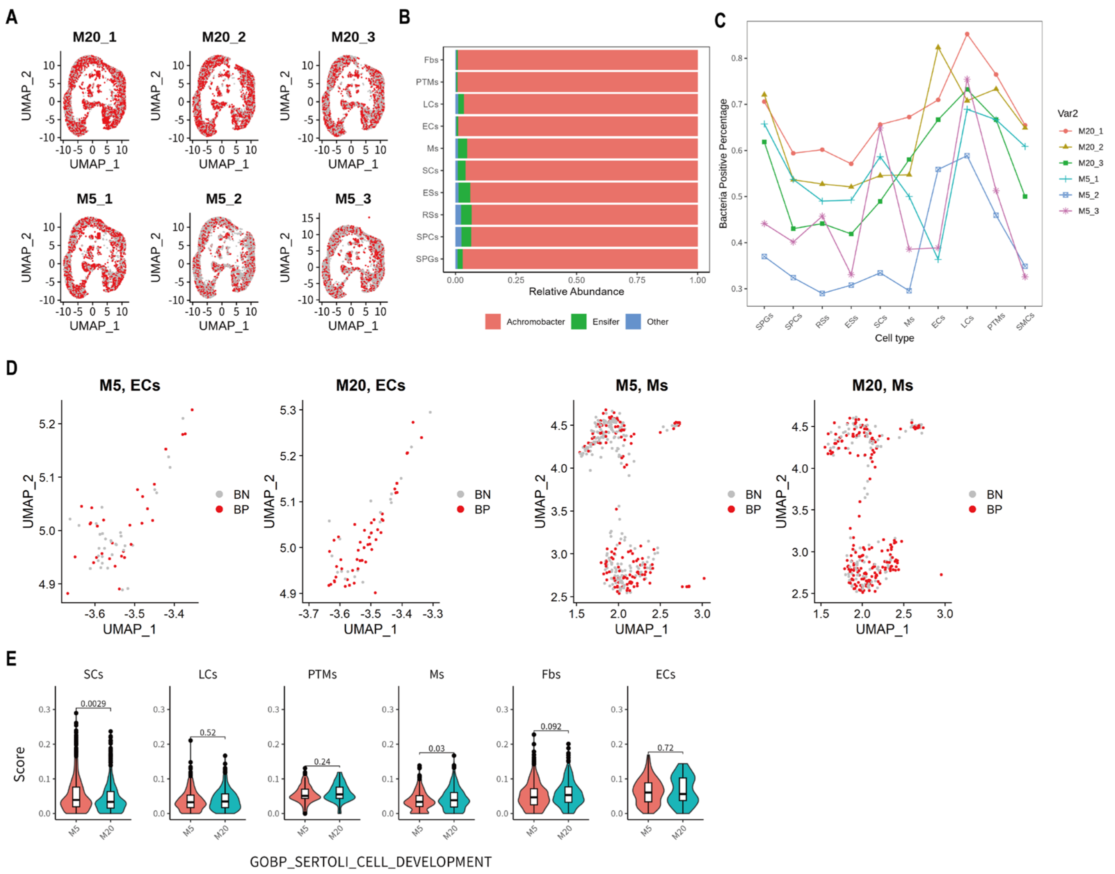

Figure S3. Single-cell analysis of testicular cells focusing on microbial associations and cell type-specific distributions from young and aged mice

A) UMAP plots illustrating the single-cell transcriptomic landscape of testicular cells for sub-samples. Cells are colored by two states, BN (bacterial-negative, gray) and BP (bacterial-positive, red).

B) Bar plot showing the relative abundance of different microbial genera in various testicular cell types.

C) Line graph depicting the bacterial positive percentage across different cell types in young and old samples.

D) UMAP plots of endothelial cells (ECs) and macrophages (Ms) from young and old samples, with cells colored by bacterial status (BN: bacterial-negative, gray; BP: bacterial-positive, red).

E) Violin plots of gene set scores for ‘sertoli cell development’ pathway across different testicular cell types in young (red) and old (blue) samples.

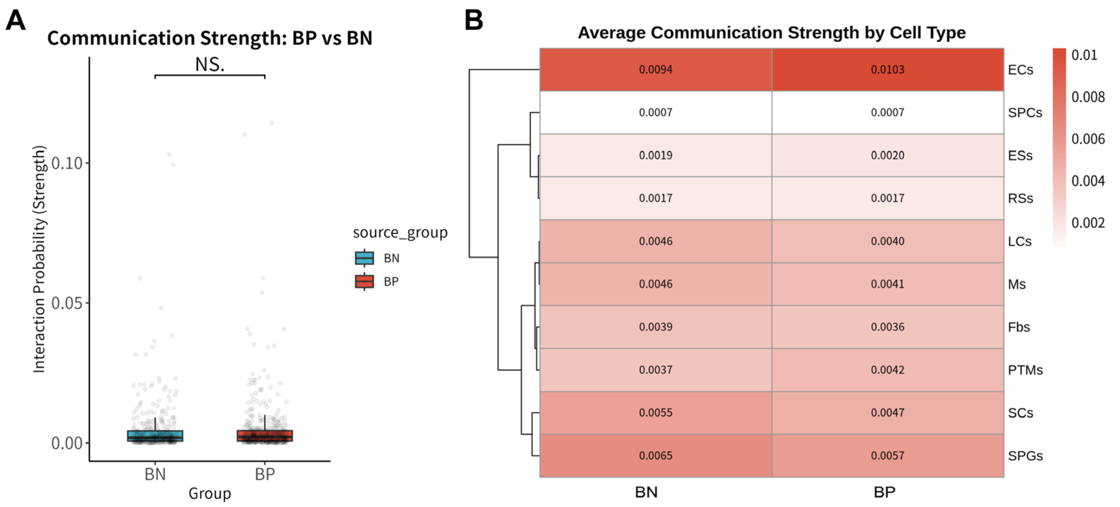

Figure S4. Cell-type-specific analysis of intercellular communication strength in bacterial-positive and bacterial-negative testicular cell populations

A) Box plot comparing the distribution of intercellular communication strength between BN (blue) and BP (red) groups. “NS.” indicates no significant difference in overall communication strength between the two groups, suggesting a lack of global alteration in intercellular signaling strength driven by bacterial presence.

B) Heatmap of average intercellular communication strength by cell type in BN and BP groups. Rows represent testicular cell types, and columns denote the groups (BN and BP). Color intensity reflects average communication strength, with a gradient from low (light) to high (dark red).

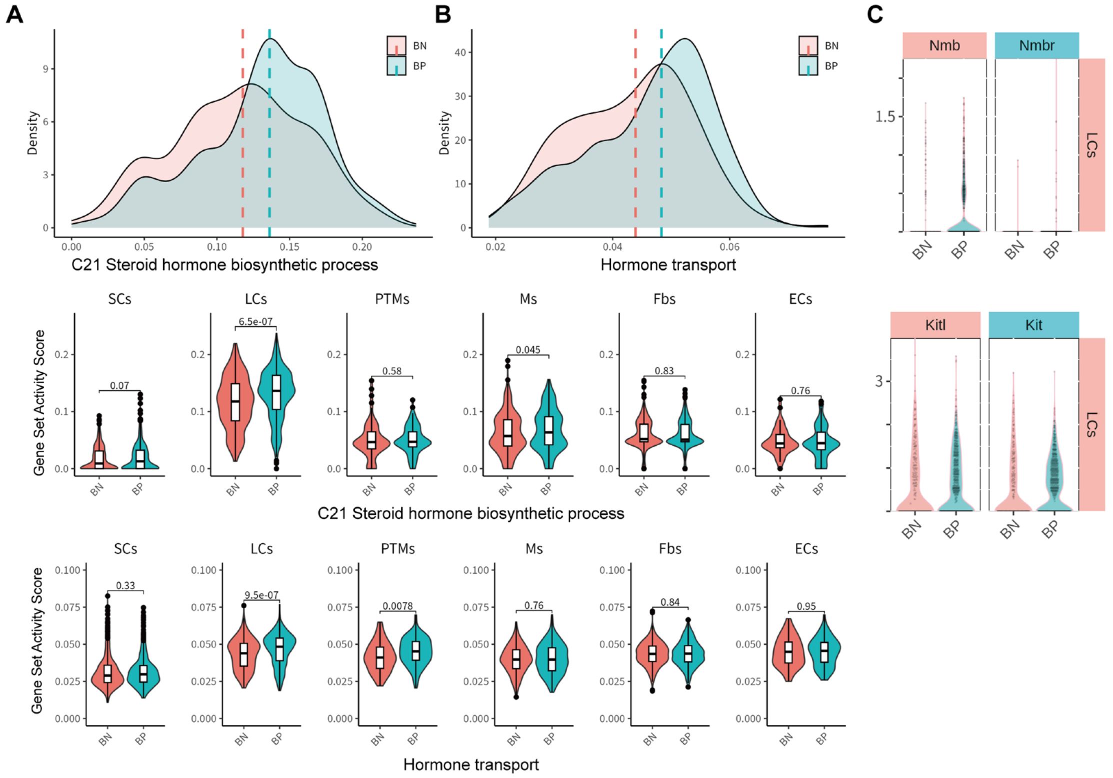

Figure S5. Bacterial presence modulates hormone-related pathways in testicular cell populations

A) Density plots (top) and violin plots (bottom) visualizing the distribution of gene set score associated with ‘C21 steroid hormone biosynthetic process’ in BN (red) and BP (blue) groups.

B) Density plots (top) and violin plots (bottom) visualizing the distribution of gene set score associated with ‘Hormone transport’ in BN (red) and BP (blue) groups.

C) Violin plots showing expression of GPR and KIT signaling genes inferred by CellChat in leydig cells (LCs).

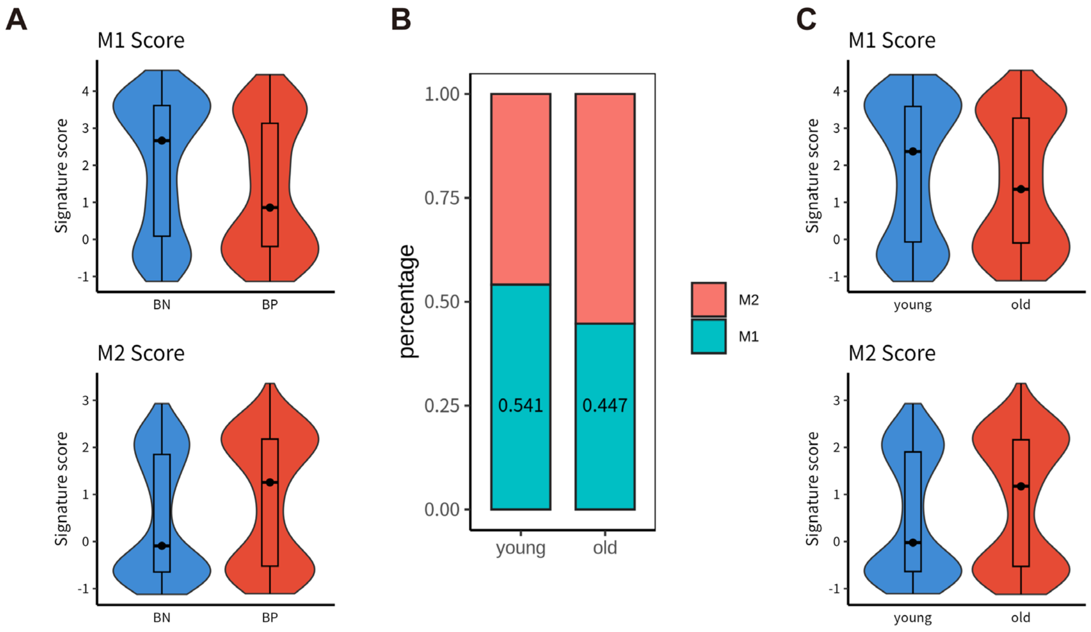

Figure S6. Comparative analysis of macrophage polarization states in relation to bacterial status and aging.

A) Violin plots showing the distribution of M1 and M2 gene signature scores in bacterial-negative (BN) and bacterial-positive (BP) macrophages. Bacterial-positive macrophages display lower M1 and higher M2 scores, suggesting a tendency toward M2-like polarization. M1 and M2 signature scores were computed using the *AddModuleScore()* function in Seurat based on curated marker gene sets derived from published macrophage polarization studies. M1 marker genes: *Cd74, H2-Aa, H2-Ab1, H2-Eb1, Ccl4, Nfkbia*; M2 marker genes: *F13a1, Ccl8, Apoe, Selenop, Cd63, Stab1*. The module score for each signature represents the average normalized expression of marker genes minus the aggregated expression of control gene sets. Higher scores indicate stronger transcriptional similarity to the corresponding macrophage polarization state.

B) Stacked bar chart comparing the proportions of M1- and M2-like macrophages in young (5-month) and old (20-month) mouse testes. Aged testes show a relatively higher fraction of M2-like macrophages.

C) Violin plots of M1 and M2 signature scores across macrophages from young and aged testes. Aged macrophages show a mild upward shift in M2 signature scores, consistent with increased bacterial abundance and enhanced M2 polarization.
